# Within-host evolution of drug tolerance in *Mycobacterium tuberculosis*

**DOI:** 10.1093/jacamr/dlag007

**Published:** 2026-02-10

**Authors:** Valerie F A March, Kakha Mchedlishvili, Galo A Goig, Nino Maghradze, Teona Avaliani, Rusudan Aspindzelashvili, Zaza Avaliani, Maia Kipiani, Nestani Tukvadze, Levan Jugheli, Selim Bouaouina, Anna Doetsch, Sevda Kalkan, Miriam Reinhard, Sebastien Gagneux, Sonia Borrell

**Affiliations:** Department of Research, Swiss Tropical and Public Health Institute, Allschwil, Switzerland; University of Basel, Basel, Switzerland; Department of Research, Swiss Tropical and Public Health Institute, Allschwil, Switzerland; University of Basel, Basel, Switzerland; National Center for Tuberculosis and Lung Diseases (NCTLD), Tbilisi, Georgia; Department of Research, Swiss Tropical and Public Health Institute, Allschwil, Switzerland; University of Basel, Basel, Switzerland; Department of Research, Swiss Tropical and Public Health Institute, Allschwil, Switzerland; University of Basel, Basel, Switzerland; National Center for Tuberculosis and Lung Diseases (NCTLD), Tbilisi, Georgia; National Center for Tuberculosis and Lung Diseases (NCTLD), Tbilisi, Georgia; National Center for Tuberculosis and Lung Diseases (NCTLD), Tbilisi, Georgia; National Center for Tuberculosis and Lung Diseases (NCTLD), Tbilisi, Georgia; European University, Tbilisi, Georgia; National Center for Tuberculosis and Lung Diseases (NCTLD), Tbilisi, Georgia; David Tvildiani Medical University (DTMU), Tbilisi, Georgia; The University of Georgia, Tbilisi, Georgia; Department of Research, Swiss Tropical and Public Health Institute, Allschwil, Switzerland; University of Basel, Basel, Switzerland; National Center for Tuberculosis and Lung Diseases (NCTLD), Tbilisi, Georgia; Department of Research, Swiss Tropical and Public Health Institute, Allschwil, Switzerland; University of Basel, Basel, Switzerland; National Center for Tuberculosis and Lung Diseases (NCTLD), Tbilisi, Georgia; Department of Research, Swiss Tropical and Public Health Institute, Allschwil, Switzerland; University of Basel, Basel, Switzerland; Department of Research, Swiss Tropical and Public Health Institute, Allschwil, Switzerland; University of Basel, Basel, Switzerland; Department of Research, Swiss Tropical and Public Health Institute, Allschwil, Switzerland; University of Basel, Basel, Switzerland; Department of Research, Swiss Tropical and Public Health Institute, Allschwil, Switzerland; University of Basel, Basel, Switzerland; Department of Research, Swiss Tropical and Public Health Institute, Allschwil, Switzerland; University of Basel, Basel, Switzerland; Department of Research, Swiss Tropical and Public Health Institute, Allschwil, Switzerland; University of Basel, Basel, Switzerland

## Abstract

**Background and objectives:**

*Mycobacterium tuberculosis* (Mtb) causes tuberculosis (TB) in humans. Poor treatment responses are a threat to global TB control, as such, understanding contributing factors to poor responses is important. We proposed that antibiotic tolerance could contribute to delayed culture conversion (recalcitrant TB), and resistance amplification in patients during TB treatment. We thus ventured to investigate the role of drug tolerance in delayed culture conversion and resistance amplification in TB patients.

**Methods:**

We collected serial Mtb isolates from patients with (i) drug-susceptible TB who remained culture positive for up to 6 years (i.e. recalcitrant TB), and (ii) multidrug-resistant TB (MDR-TB) where resistance amplified during treatment. We measured tolerance to rifampicin in drug-susceptible TB strains and tolerance to moxifloxacin in MDR-TB strains using a real-time time–kill assay.

**Results and discussion:**

Rifampicin tolerance evolved within-host, increasing up to and ∼1.5-fold, however, there was no apparent contribution of rifampicin tolerance to delayed culture conversion. Tolerance to moxifloxacin in MDR-TB patients appeared negatively associated with resistance amplification and consistently decreased over time in patients.

**Conclusion:**

Our findings confirm that antibiotic tolerance evolves in Mtb within patients over time during treatment. However, there was no evidence that this tolerance influences treatment responses, calling for further investigation of contributors to adverse treatment responses and their mitigation.

## Introduction

With a reported ∼1.2 million tuberculosis (TB) related deaths in 2023, *Mycobacterium tuberculosis* (Mtb) is the deadliest human infectious agent.^1,2^ The standard of care to treat TB is combination antibiotic chemotherapy, which is highly efficacious for drug-susceptible TB (DS-TB),^1^ however, drug resistance emerges and threatens TB control.

Multidrug-resistant TB (MDR-TB) characterized by resistance to frontline drugs rifampicin and isoniazid, has historically been less successfully treated.^1^ Regimens to treat MDR-TB include second-line agents such as fluoroquinolones^3–5^ and now incorporate novel anti-TB drugs,^6–8^ for which, alarmingly, strains harbouring resistance conferring mutations are already circulating.^9^ Resistance amplification, where drug-resistant strains gain further drug resistance, complicates patient treatment as there are limited efficacious second-line agents available.

Studies have shown that TB treatment outcomes are affected by sociodemographic factors such as sex and age,^10–19^ alongside comorbidities such as HIV coinfection^11,13,15,17,18,20,21^ or having a history of TB.^10,12–14,16,22^ In addition, poor responses to treatment such as delayed culture conversion (failure to culture convert after 2–3 months)^10,14^ as well as drug resistance^23,24^ increase the risk of poor treatment outcomes in TB. Recently, we published a study using clinical and bacterial genomic data from Georgia, demonstrating bacterial determinants can contribute to treatment outcomes,^25^ thus setting a precedent to further explore the contribution of bacterial determinants to treatment outcomes.

In other diseases, drug tolerance, defined as the ability of bacteria to withstand extended durations in bactericidal antibiotics,^26,27^ has been demonstrated to contribute to chronic infection in patients^28^ and the emergence of antibiotic resistance.^29^ The impact of tolerance in TB has been underexplored, despite being a phenotype that can develop within patients even under combination therapy.^30^ As highlighted by Deventer and colleagues, there is minimal research exploring the clinical impact of drug tolerance, with much of our understanding coming from *Staphylococcus aureus* research, indicating a research gap.^27^

With access to serial Mtb clinical isolates from historical TB patients at the National Centre for Tuberculosis and Lung Disease (NCTLD) in Tbilisi, Georgia, we investigated the hypothesis that antibiotic tolerance can contribute to poor treatment responses in TB. We examined the contribution of tolerance to rifampicin and fluoroquinolone moxifloxacin to delayed culture conversion (recalcitrant TB) and resistance amplification in strains from DS-TB and MDR-TB patients, respectively.

## Methods

### Ethical approval

The Institutional Review Board of the NCTLD in Tbilisi, Georgia and the Ethics Commission of North- and Central Switzerland granted ethical approval for the use of the patient samples and data used in this study. The ethics committees waived the need for individual patient consent since only limited and anonymized clinical data were used.

### Strain selection

We explored a retrospective database of laboratory clinical data from Georgian active pulmonary TB patients spanning the period between January 2008 and December 2022. We selected patients with multiple serial Mtb isolates with phenotypic drug susceptibility data that fit into one of four groups: DS-TB control (Group A, *n* = 3), DS-TB recalcitrant (Group B *n* = 6), MDR-TB control (Group C, *n* = 4) and MDR-TB resistance amplification (Group D, *n* = 5). All patients were diagnosed, monitored and treated according to the Georgian National TB Program, which includes molecular and culture-based drug susceptibility testing (DST).^31^ Group A patients were initially diagnosed with DS-TB and did not exhibit delayed culture conversion. Group B patients were also diagnosed as DS-TB but remained sputum culture positive for up to 6 years despite receiving treatment, without drug resistance evolving. Group C patients were diagnosed with MDR-TB and remained classified as such throughout treatment. Group D patients were diagnosed with MDR-TB and gained additional resistance during treatment. Our resulting cohort was comprised of strains from 18 bacteriologically confirmed adult TB patients. Strains from these patients were designated a unique identifier which was used to coordinate strain culture and DNA extraction in Georgia. After WGS analysis (available as Supplementary data at *JAC-AMR* Online) to confirm that phenotypic DST and genotypic DST prediction were concordant, strains were shipped to our BLSL3 facilities in Switzerland for further processing and experiments.

### Preparation of antibiotic stocks

Antibiotic stocks were prepared by dissolving powdered stocks of rifampicin (Sigma-Aldrich) and moxifloxacin (Sigma-Aldrich) in dimethyl sulfoxide (DMSO, Applichem) to a desired stock concentration. The same drug stocks were used for MIC measurements and time–kill assays.

### Measuring antibiotic tolerance

Tolerance to rifampicin was measured for drug-susceptible strains and tolerance to moxifloxacin was measured for MDR strains using our real-time time–kill assay, as previously described.^31^ In short, 50 µL of thawed calibrated stocks of strains were inoculated into 96-well u-bottom deep-well plates (Thermo Fisher) containing 950 µL of 7H9 ADC with or without antibiotic at 400× MIC in triplicate. Resulting cultures were mixed thoroughly before 100 µL was sampled for viability using the BacTiter-Glo assay in a 1:1 ratio. BacTiter-Glo plates were incubated for 30–35 minutes before measuring luminescence using the Tecan Spark Multimodal plate reader for susceptible strains and the Tecan Infinite Pro 200 for MDR strains. Killing was measured every 4 days for 14 days, with a final measurement at 21 days post-inoculation. For moxifloxacin experiments, a measurement after 2 days was included.

After controlling for background luminescence by subtracting signal from culture-free negative controls, killing was assessed by calculating the fraction of survival for each time point by dividing the signal measured in drug conditions with the initial luminescence of the untreated control. The minimum duration for killing (MDK) for each strain was determined by using the resulting survival data in our in-house MDK calculator (GetMDK, https://git.scicore.unibas.ch/TBRU/mdkcalculator).^31^ MDK thresholds were adjusted to maximize the number of strains that could be included in downstream analyses. For moxifloxacin-treated strains, a threshold of 90% was used due to a strain exhibiting extensively high tolerance compared with others [which had minimal impact on our conclusions, see Figure S5 (available as Supplementary data at *JAC-AMR* Online)], whereas a threshold of 98% was used for rifampicin-treated strains.

### Statistical analysis

Statistical analysis and data visualization were performed using GraphPad Prism version 8.2.1. Tolerance and MIC data were imported into the Prism program and visualized. Data were tested for normality and *t*-tests were used to compare data between groups; otherwise, non-parametric equivalents were used. Specific statistical tests are specified in figure captions.

## Results and discussion

### A cohort of TB patients with diverse clinical outcomes

**Figure 1.**
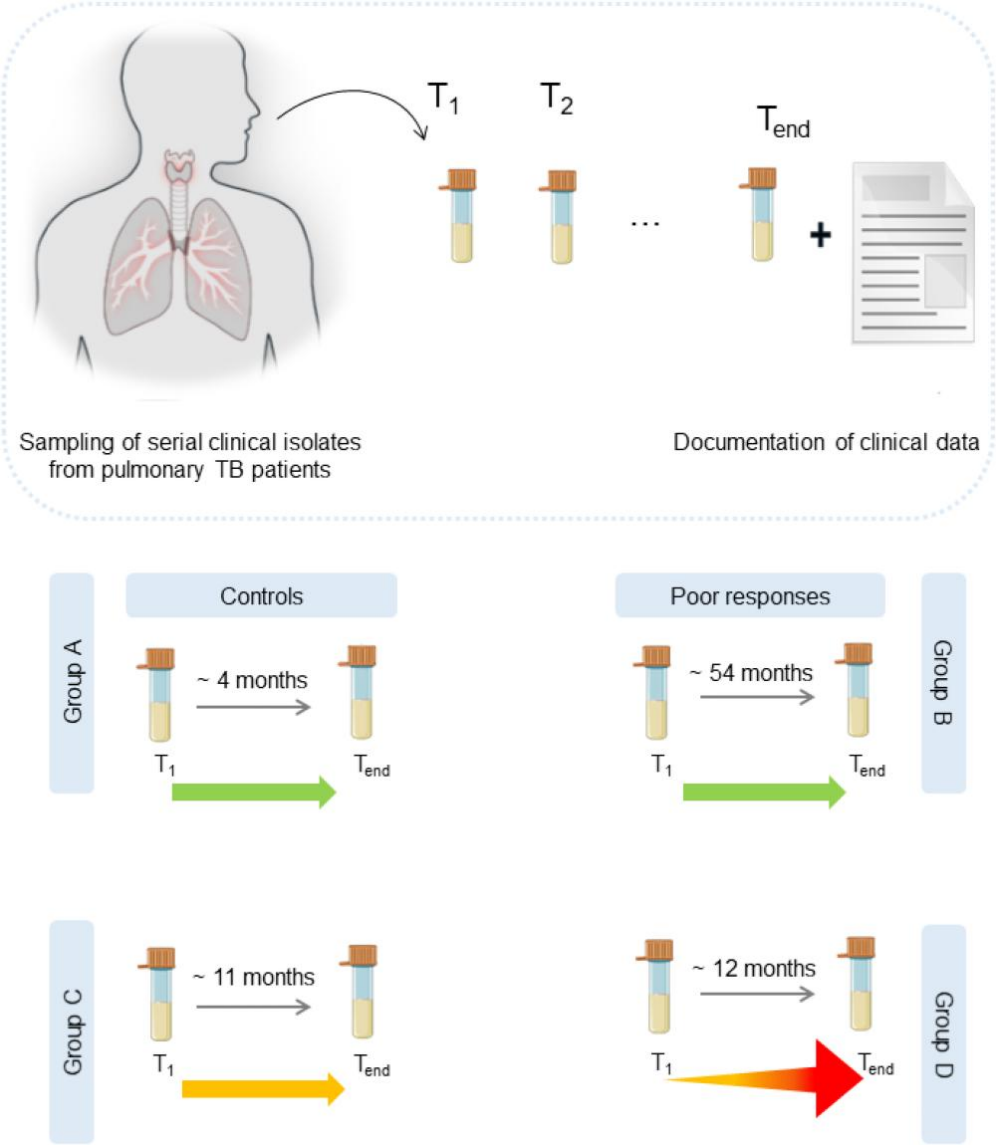
Scheme of clinical isolate origin and strain classification. Serial Mtb isolates were collected from TB patients throughout their TB episodes. Initial (*T*_1_) and final (*T*_end_) isolates from patients were selected and categorized into groups according to drug susceptibility and patient responses. Time span between isolates in diagram represent median time span of isolates within the group. Group A strains (*n* = 3 strains and patients) were phenotypically drug susceptible and were cured within 13 months. Group B strains (*n* = 12) were drug susceptible and remained so, but patients (*n* = 6) had delayed culture conversion. Group C strains (*n* = 8 strains, *n* = 4 patients) were MDR and remained so until final sampling. Group D strains (*n* = 5) were initially MDR and patients (*n* = 5) experienced drug resistance amplification.

We selected a cohort of 18 patients and 28 strains, to interrogate the clinical relevance of antibiotic tolerance in TB (Figure 1, Table 1).

**Table 1.** Strain information

Strain	Patient	Isolate no./total	Timespan of isolates (months)	DST	Lineage	Category
N50209	P01	1/8	56.1	Susceptible	L2	Group B
N50215	P01	8/8		Susceptible	L2	Group B
N50225	P02	1/9	75.1	Susceptible	L4	Group B
N50232	P02	8/9		Susceptible	L4	Group B
N50247	P03	1/8	52.5	Susceptible	L4	Group B
N50255	P03	8/8		Susceptible	L4	Group B
N50323	P04	1/5	79.2	Susceptible	L4	Group B
N50327	P04	5/5		Susceptible	L4	Group B
N50339	P05	1/9	22.1	Susceptible	L4	Group B
N50246	P05	9/9		Susceptible	L4	Group B
N50183	P06	1/8	53.5	Susceptible	L4	Group B
N50189	P06	7/8		Susceptible	L4	Group B
N50445	P07	1/4	12.8	Susceptible	L4	Group A
N50447	P08	1/4	2.1	Susceptible	L4	Group A
N50450	P09	1/4	3.9	Susceptible	L4	Group A
N50180	P10	1/4	7.5	MDR	L2	Group C
N50182	P10	4/4		MDR	L2	Group C
N50262	P11	1/5	14	MDR	L2	Group C
N50265	P11	5/5		MDR	L2	Group C
N50335	P12	1/4	8.7	MDR	L4	Group C
N50338	P12	4/4		MDR	L4	Group C
N50238	P13	1/6	81.6	MDR	L2	Group C
N50241	P13	4/6		MDR	L2	Group C
N50205	P14	1/4	31.03	MDR	L2	Group D
N50267	P15	2/3	1.9	MDR	L2	Group D
N50269	P16	1/5	17.1	MDR	L2	Group D
N50233	P17	1/4	5.1	MDR	L2	Group D
N50278	P18	1/5	12.5	MDR	L2	Group D

To confirm that patients were infected with the same genotype throughout the course of their episode and to genotypically monitor drug resistance markers, we performed whole-genome analysis on all isolates. From our phylogenetic analysis, we saw that isolates from the same patient separated in time clustered together (Figure S1) with SNP distances ranging from 2–16 SNPs (Figure S1), supporting the notion that they were effectively the same strain in line with previously reported SNP distances for epidemiologically linked isolates.^32–34^ MDR isolates were mainly members of *Mycobacterium tuberculosis* complex lineage (L) 2, whereas drug-susceptible isolates were mainly members of L4 which corresponds with general Georgian TB molecular epidemiology^25,35,36^; as such, our sample set was endemically relevant.

### Tolerance does not explain poor treatment responses

**Figure 2.**
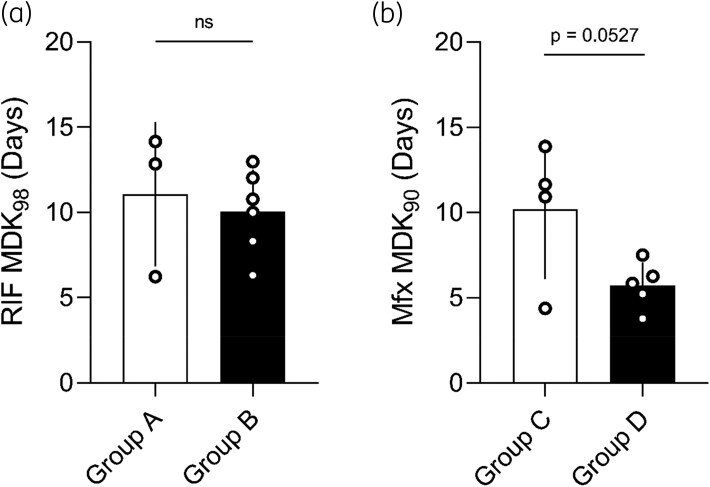
Differences in rifampicin tolerance do not account for recalcitrant TB, but low moxifloxacin tolerance may play a role in resistance amplification. (a) Comparison of rifampicin (RIF) tolerance in strains isolated from control versus recalcitrant susceptible patients. Bars denote mean, error bars indicate standard deviation, ns = not significant by unpaired *t-*test. (b) Comparison of tolerance to moxifloxacin (Mfx) in strains isolated from control versus resistance amplification patients. Bars denote mean, error bars indicate standard deviation, *P* = 0.0527 by unpaired *t*-test.

While there has been evidence that tolerance exists and varies in Mtb,^37,38^ its clinical relevance has not been thoroughly established. To investigate whether Group B cases were more tolerant to rifampicin and thus remained culture positive without gaining resistance, we measured rifampicin tolerance in Group A cases compared with Group B cases. We found no significant differences in rifampicin MDK_98_ values between strains from Group A and Group B patients (Figure 2a). In addition, rifampicin MICs were relatively stable (Figures S2 and S3), thus rifampicin susceptibility could not explain recalcitrant TB in our strain set.

To explore whether moxifloxacin tolerance is involved in resistance amplification in MDR-TB, we compared moxifloxacin tolerance between Group C and Group D strains. Contrary to what has previously been reported in other bacteria, we saw lower moxifloxacin MDK_90_ values in Group D strains compared with Group C. While this difference did not reach formal statistical significance (Figure 2b, *P* = 0.0527), the strains that eventually gained fluoroquinolone resistance to become pre-XDR by definition,^6^ had lower moxifloxacin tolerance compared with those that remained MDR. In contrast, moxifloxacin MICs had no statistically significant difference between Group C and D (Figure S4). The median time interval between initial and final isolate between Group C and D patients was similar (∼11 and 12 months, Figure 1), showing that within the same time-frame, some strains experienced resistance amplification while others did not.

In other bacteria, tolerance has been shown to lead to resistance evolution.^28–30^ Specifically, if two strains with equal drug susceptibility undergo a protocol of high-dose intermittent exposure to antibiotics, the strain that started more tolerant would evolve drug resistance sooner. In our data, lower Mfx tolerance was associated with resistance amplification, which goes against this convention and requires further exploration. However, under both drug conditions, tolerance varied among strains (Figure S6), indicating that it is an adaptable trait.

### Within-host evolution of tolerance has different trajectories

TB treatment requires a minimum of 6 months of antibiotic chemotherapy, which could allow for within-host adaptation of Mtb. Serial isolates provide an opportunity to observe such adaptation across time. Having analysed the initial and final isolates from Group B and C patients, we were able to evaluate the dynamics of tolerance across time within-host.

**Figure 3.**
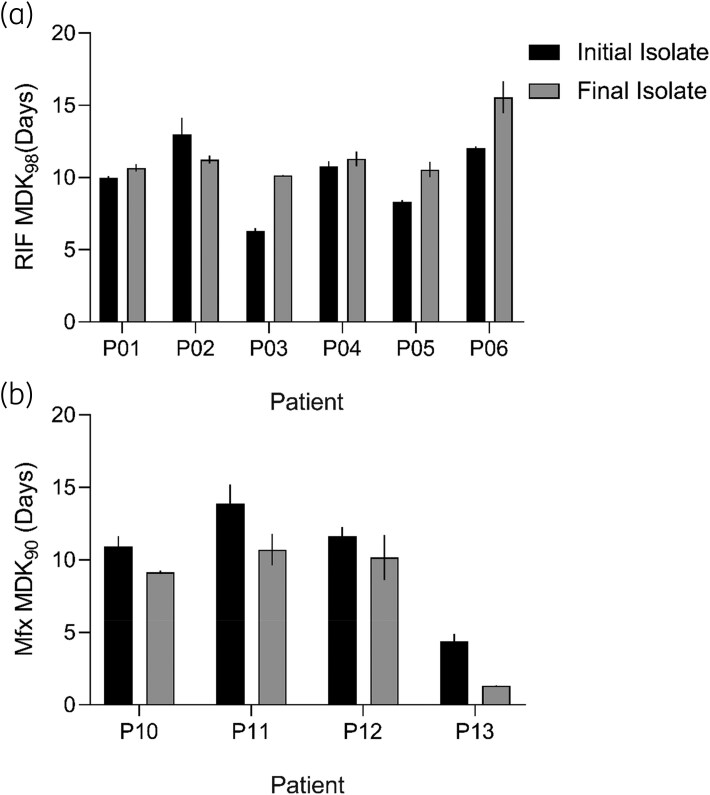
Within-host of dynamic of tolerance in TB patients. (a) Comparison of change in tolerance rifampicin (RIF) between initial and final isolates from Group B patients. (b) Comparison of changes in moxifloxacin (Mfx) tolerance in Group C patients. Bars denote a mean of *n* = 2 independent experiments; error bars denote standard error.

The change in rifampicin tolerance was heterogeneous across isolates from Group B patients, in that tolerance increased (P03, P05, P06), remained stable (P01 and P04) or decreased (P04) across time (Figure 3a). The direction of the change in RIF tolerance did not appear to be related to the time interval between isolates (Table 1). The increase in RIF tolerance was notably higher by ∼1.6-fold and ∼1.3-fold in P03 and P06 final isolates, respectively, compared with their initial isolates (Figure 3a). This could indicate that higher RIF tolerance could be an effect of delayed culture conversion rather than a cause as we had initially expected, or as reported in other bacteria.^30^ This could be due to poor treatment adherence or insufficient treatment as in patients P03 and P06 who are both retreatment cases, thus representing intermittent drug exposure for the bacteria inside the host. *In vitro*, intermittent exposure to antibiotics evolves drug tolerance,^28,30^ as such, intermittent treatment is a plausible contributing factor to the evolution of rifampicin tolerance in TB patients.

In Group C, final isolates consistently exhibited lower moxifloxacin MDK_90_ values and thus tolerance compared with the initial isolate, both within-host (Figure 3b), and among hosts (Figure S7). This finding indicates a tendency for Mfx tolerance to decrease over time in Mtb in patients undergoing MDR-TB treatment. This subtle evidence of a reversal of drug tolerance over time is unprecedented, but the decrease was consistent among all patients, stoking a need for further investigation.

### Conclusion

Here we explored antibiotic tolerance in Mtb isolates from TB patients with defined treatment responses with respect to recalcitrance and resistance amplification. Our work has several limitations. First, our patient sample sizes are small. Despite this, we have been able to observe different manifestations of the dynamics of within-host drug tolerance. Second, due to the age of some of our Mtb isolates, some patient data was irretrievable. More complete clinical data could shed further light on where these dynamic changes in tolerance clinically manifest. Finally, while we set out to understand bacterial determinants that contribute to poor outcomes, we neglected to consider patient features within the drug–pathogen interaction. Patients can differ in their metabolism of TB drugs,^39,40^ which could result in bacteria experiencing sub-lethal doses, thereby influencing culture conversion and acquired drug resistance.^41^ While we did not have access to patient pharmacokinetics and pharmacodynamics, which also affect treatment efficacy,^34,42,43^ in our historical data, future prospective studies endeavouring to understand within-host drug-shaped Mtb evolution, should consider this.

In conclusion, we provide evidence that antibiotic tolerance changes within-host in a patient and drug-specific manner, with an unclear contribution to patient treatment outcomes. Which is in line with current understanding of the contribution of drug tolerance to clinical outcomes in patients.^27^ However, it is likely that drug susceptibility by way of drug tolerance, alongside host-derived features, work together in contributing to treatment responses, and as such should be further evaluated in larger sample sets.

## Supplementary Material

dlag007_Supplementary_Data

## Supplementary data

Figures S1 to S7 are available as Supplementary data at *JAC-AMR* Online.
